# Artificial Blood for Dogs

**DOI:** 10.1038/srep36782

**Published:** 2016-11-10

**Authors:** Kana Yamada, Kyoko Yokomaku, Moeka Kureishi, Motofusa Akiyama, Kiyohito Kihira, Teruyuki Komatsu

**Affiliations:** 1 Department of Applied Chemistry, Faculty of Science and Engineering, Chuo University, 1-13-27 Kasuga, Bunkyo-ku, Tokyo 112-8551, Japan; 2 JEM Utilization Center, Human Spaceflight Technology Directorate, Japan Aerospace Exploration Agency (JAXA), 2-1-1 Sengen, Tsukuba-shi, Ibaraki 305-8505, Japan

## Abstract

There is no blood bank for pet animals. Consequently, veterinarians themselves must obtain “blood” for transfusion therapy. Among the blood components, serum albumin and red blood cells (RBCs) are particularly important to save lives. This paper reports the synthesis, structure, and properties of artificial blood for the exclusive use of dogs. First, recombinant canine serum albumin (rCSA) was produced using genetic engineering with *Pichia* yeast. The proteins showed identical features to those of the native CSA derived from canine plasma. Furthermore, we ascertained the crystal structure of rCSA at 3.2 Å resolution. Pure rCSA can be used widely for numerous clinical and pharmaceutical applications. Second, hemoglobin wrapped covalently with rCSA, hemoglobin–albumin cluster (Hb-rCSA_3_), was synthesized as an artificial O_2_-carrier for the RBC substitute. This cluster possesses satisfactorily negative surface net charge (*p*I = 4.7), which supports enfolding of the Hb core by rCSA shells. The anti-CSA antibody recognized the rCSA exterior quantitatively. The O_2_-binding affinity was high (*P*_50_ = 9 Torr) compared to that of the native Hb. The Hb-rCSA_*3*_ cluster is anticipated for use as an alternative material for RBC transfusion, and as an O_2_ therapeutic reagent that can be exploited in various veterinary medicine situations.

Japan has become “a pet superpower”. Official estimates put the pet population at 19,791,000 (9,917,000 dogs and 9,874,000 cats), which is far greater than the number of young people under the age of 15 (at 16,040,000)^1^,^2^. In the country, the human birthrate has been gradually falling while the average age of the population has been rapidly climbing. Pet animals, which can enrich human life, are now treated as family members. As one might expect, the demand for pet medical care continues to grow, and the frequency of “blood transfusion” continues to increase. Nevertheless, the shortage of blood products, especially for dogs, is little recognized. Because blood banks for pet animals are not approved in Japan, veterinarians themselves must obtain blood for transfusion therapy of hemorrhagic-shocked or anemic patients. The most difficult problem is how to find a donor. Among blood components, serum albumin and red blood cell (RBC) are particularly important to save lives. The challenges confronting the present study were two-fold. First, a recombinant canine serum albumin (rCSA) was produced using genetic engineering procedures. In the mammalian circulatory system, serum albumin is the most abundant protein (typical concentration of 2–5 g/dL). It plays two major roles of (i) transporting diverse metabolites and drugs and (ii) maintaining colloid osmotic pressure of the bloodstream^3^,^4^. Valenta *et al*. reported the expression of rCSA using *E. coli*, although the quantity of the protein was insufficient to explore physicochemical features^5^. Some concern also persists about the contamination of toxic lipopolysaccharide by *E. coli* membranes. If it were possible to produce rCSA using *Pichia* yeast as a host cell, then rCSA could be manufactured to prepare a plasma expander for clinical use and a new formulation for pharmaceutical and biochemical applications. We have established the efficient *Pichia pastoris* expression system of rCSA, and defined its physicochemical properties. Moreover, X-ray crystallography has revealed the three-dimensional (3D) structure of the protein at 3.2 Å resolution.

A second challenge is the creation of artificial O_2_-carrier as an RBC substitute. Naturally, dogs have different blood types. For example, dog erythrocyte antigen (DEA) classification divides blood types into nine kinds (DEA1.1, 1.2, 1.3, 3, 4, 5, 6, 7, and 8)^6^. The blood of DEA1.1-negative donor can be transfused into any dog. On the one hand, transfusion of the DEA1.1-positive donor’s blood into a DEA1.1-negative recipient generates an antibody. Consequently, a second infusion of the DEA1.1-positive type blood will cause an acute hemolytic reaction. To conduct safe blood transfusion, it is necessary to confirm blood compatibility in advance. Of course, veterinarians would not need to find a donor and test blood compatibility before infusion if an RBC substitute for dogs were realized.

Studies of RBC substitutes for human use have continued since the 1980s^7^,^8^,^9^,^10^. The products with the greatest potential are hemoglobin (Hb)-based O_2_ carriers^9^,^10^. In the United States, poly(ethyleneglycol)-conjugated Hb^11^,^12^,^13^, polymerized Hb^14^, intramolecularly cross-linked Hb^15^,^16^ have been developed and examined in clinical trials^7^,^8^,^9^,^10^,^17^,^18^,^19^,^20^. Most of these artificial O_2_-carriers are designed to increase molecular size and to avoid renal excretion. However, transfusion of such heterogeneous molecules has raised the blood pressure in humans to a greater or lesser extent. Two mechanisms for this side effect are presumed to be (i) nitric oxide (NO: an endothelium-derived relaxing factor) scavenging by Hb diffused into the extravascular space and (ii) oversupply of O_2_ to vascular walls by facilitated diffusion^8^,^17^,^18^,^19^,^20^,^21^,^22^,^23^,^24^. Despite intensive efforts, no substitute has been able to satisfy all requirements for clinical use. For pet animals, polymerized bovine Hb (Oxyglobin^®^) used to be commercially available for the treatment of anemic dogs in the United States and the United Kingdom^25^. Alayash *et al*. reported that this formulation contained nonpolymerized Hb of a substantial amount (37.8%)^26^. Some side effects have been reported, such as vomiting, anorexia, and pyrexia, as well as discoloration of skin, mucous membranes, urine, and (darkened) feces^27^.

We have recently synthesized a covalent core–shell structured protein cluster comprising a bovine Hb in the center and human serum albumins (HSAs) at the periphery, hemoglobin–albumin cluster (Hb-HSA_*3*_), as a unique artificial O_2_-carrier^28^,^29^,^30^,^31^. The Hb nucleus is wrapped covalently with HSA. Thereby the surface net charge of the cluster is negative to a marked degree [isoelectric point (*p*I): 5.1]. As a consequence, the Hb-HSA_*3*_ cluster shows long circulation persistence and does not engender an acute increase in blood pressure^31^. This result is ascribed to the electrostatic repulsion between the negatively charged surface of the cluster and glomerular basement membrane around the endothelial cells. To develop this product for canine use, it is necessary to replace the exterior HSA shell to CSA. Injection of the Hb-HSA_*3*_ cluster into a dog might generate antibodies in the blood^32^. The recipient will therefore experience an immunological response from the second infusion. Unfortunately, it is difficult to obtain canine blood (plasma) of sufficient quantity for the large-scale production of the cluster. To resolve this difficulty, we have used genetically engineered rCSA. A new hemoglobin–albumin cluster, Hb-rCSA_*3*_, can be regarded as a promising RBC alternate for dogs as well as an O_2_-carrying fluid that is useful in various veterinary medicine scenarios. The Hb-rCSA_*3*_ cluster has no blood type. It can be transfused into any dog at anytime, anywhere. Herein, we describe the synthesis, structure, and O_2_-binding properties of the Hb-rCSA_*3*_ cluster. The rCSA and Hb-rCSA_*3*_ cluster can contribute greatly to pet medical care.

## Results

### Expression and Physicochemical Property of rCSA

Expressions of rCSA using *Pichia* yeast have not been reported in the relevant literature. We have now prepared rCSA using *Pichia pastoris* (GS115) in a gram quantity. *Pichia* yeast secretes the target protein into culture medium so that the accumulated protein can be harvested easily by centrifugation, followed by general chromatographic technique. Results showed that the ideal purification was achieved using a combination of affinity chromatography and anion exchange chromatography.

**Figure 1:**
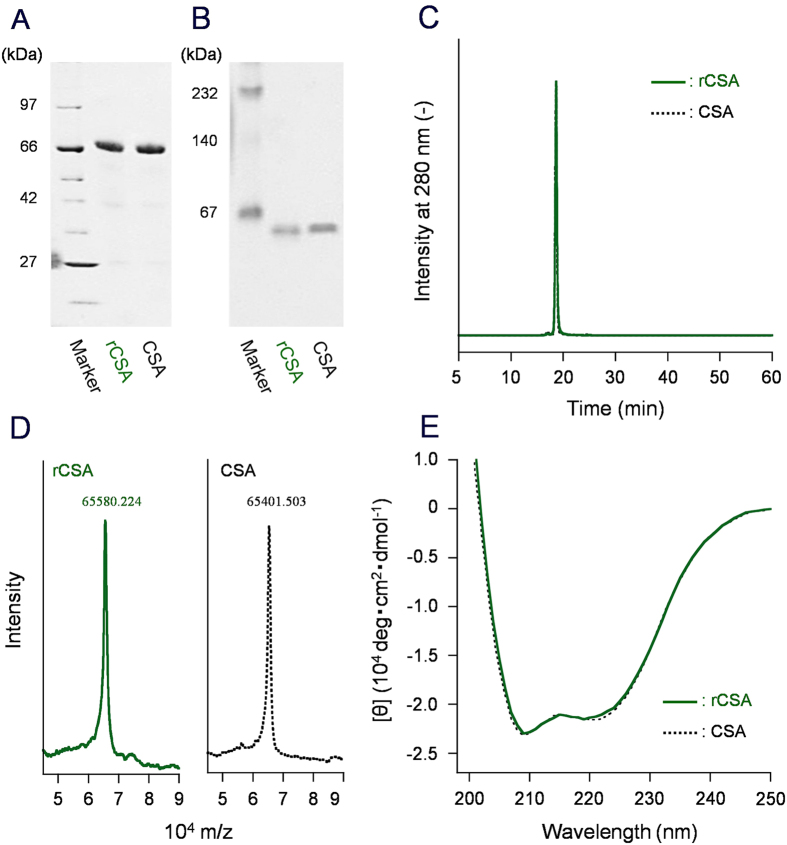
Physicochemical data of rCSA and native CSA. (**A**) SDS-PAGE, (**B**) Native-PAGE, (**C**) SEC profiles, (**D**) MALDI-TOF mass spectra, and (**E**) CD spectra of rCSA and CSA. All results demonstrated clearly that these proteins possess identical molecular weight and structure. Full-length gel images of (**A,B**) are presented in Supplementary Fig. S1(A,B).

The obtained rCSA was compared carefully to native CSA derived from canine plasma by physicochemical measurements. SDS-PAGE of rCSA exhibited a single band (Fig. 1A and S1A). Its mobility was identical to that observed for CSA. Native-PAGE also demonstrated the same result (Fig. 1B and S1B). Size-exclusion chromatography (SEC) measurements of rCSA showed a sharp and single peak that has equivalent mobility for native CSA (Fig. 1C). The rCSA purity was ascertained as 99.99%. The isoelectric focusing pattern depicted one band in both rCSA and CSA with a *p*I value of 4.5 (vide infra), which was significantly lower than that of HSA (*p*I = 5.0). Although the numbers of acidic amino acids in CSA and HSA are exactly the same (36 Asp, 62 Glu), CSA possesses fewer basic amino acids (12 His, 56 Lys, 23 Arg) relative to HSA (16 His, 59 Lys, 24 Arg)^5^,^33^. Consequently, the total charges of the rCSA molecule become negative. MALDI-TOF mass spectrum of rCSA demonstrated a molecular-related ion peak at 65580.224 Da, which was within the experimental range of the simulated mass from the sequence (65709.459 Da) and the observed mass of native CSA (65401.503 Da) (Fig. 1D). The UV-visible absorption spectrum of phosphate-buffered saline (PBS) solution (pH 7.4) of rCSA showed a single broad band at 280 nm (data not shown). The molar absorption coefficient (*ε*_280_) of rCSA was calculated as 4.50 × 10^4^ M^−1^ cm^−1^, which is almost identical to the value of CSA. The high *ε*_280_(CSA) compared to *ε*_280_(HSA) (3.50 × 10^4^ M^−1^ cm^−1^)^3^ is attributable to the existence of many Tyr residues (21 Tyr in CSA, 18 Tyr in HSA). The CD spectra of rCSA and CSA in PBS solution were indistinguishable (Fig. 1E), suggesting that their peptide backbone conformations are equivalent. All these physicochemical results imply that rCSA is structurally identical to the native CSA.

### Crystal Structure of rCSA

**Figure 2:**
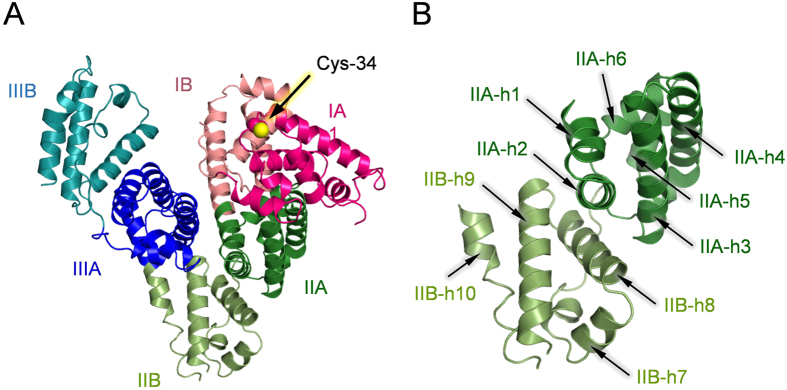
Crystal structure of rCSA. (**A**) The rCSA comprises three morphologically same domains I–III, each of which is divided into two subdomains: A and B. The Cys-34 in subdomain IA is an only Cys residue with sulfhydryl group. (**B**) Spatial alignments of α-helices in domain II. Subdomain IIA (forest) contains six helices h1–h6; subdomain IIB (pale green) contains four helices h7–h10.

The crystal structure of HSA at 2.8 Å resolution was first reported by Carter *et al*. in 1992^34^. Subsequently numerous structures not only of unliganded HSA, but also HSA complexed with specific ligands have been published by several research groups^35^,^36^,^37^,^38^. More recently, Chruszcz and Minor *et al*. have dissolved crystal structures of bovine serum albumin, equine serum albumin, and leporine serum albumin^39^. Nonetheless, the crystallographic coordinate of CSA has never been deposited in PDB data. The primary sequence of CSA indicates that the protein is a single polypeptide with 584 amino acids (Fig. S2)^5^,^33^. Pairwise sequence homology of CSA with respect to HSA is 79.8%. Our results show that the crystals of rCSA belong to the monoclinic space group P 1 21 1 (Table S1), and that the overall structure closely resembles those of HSA and other mammalian albumin (Fig. 2A and S3)^34^,^35^,^36^,^37^,^38^,^39^. The rCSA consists of three topologically identical domains (I, residues 1–196; II, residues 197–383; and III, residues 384–584), each of which is divided into two subdomains (A and B). They are composed, respectively, of six α-helices (h1–h6) and four α-helices (h7–h10) (Fig. 2B). Despite the symmetrical repeating morphology of domains I–III, their asymmetric spatial alignments form a unique heart-shape structure. As described above, rCSA has fewer basic amino acid residues than HSA has, and also more non-polar amino acid residues. The relative acidic property of rCSA was more clearly visible by its electrostatic potential representation than HSA was (Fig. S4).

There are total of 35 Cys residues in rCSA. The Cys-34 in subdomain IA is the only Cys having a free sulfhydryl group which does not participate in a 17 disulfide bridges. In the crystal structure of HSA (PDB ID: 1E78)^36^, it is apparent that the S atom of Cys-34 is surrounded by a flexible loop (Val-77–Tyr-84; sequence VATLRETY) that prevents the sulfhydryl group from coupling with an external thiol-containing compound (glutathione or cysteine). In terms of the general structure, the environment around the Cys-34 of rCSA has similar features to those of HSA. However, three differences are apparent in the amino acid sequence of the loop in rCSA; VASLRDKY containing shorter Ser, Asp, and positively charged Lys. We inferred that the space around the S atom of Cys-34 in rCSA is slightly flexible and more polar than that of HSA. This finding is consistent with the fact that the mercapto-ratio (free sulfhydryl group ratio) of the Cys-34 residue in rCSA (ca. 30–40%) was lower than the value of HSA (ca. 50–60%).

### Synthesis of Hb-rCSA_*3*_ Cluster

**Figure 3:**
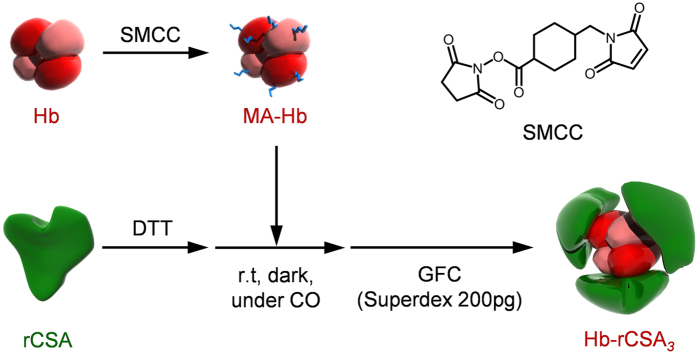
Schematic illustration of the synthetic route of Hb-rCSA_*3*_ cluster. The Cys-34 of rCSA and the surface Lys group of Hb were connected covalently with a cross-linking agent (SMCC). The product is a mixture of Hb-rCSA_m_ (m = 2, 3, 4). The total yield based on the Hb concentration was 75%.

**Figure 4:**
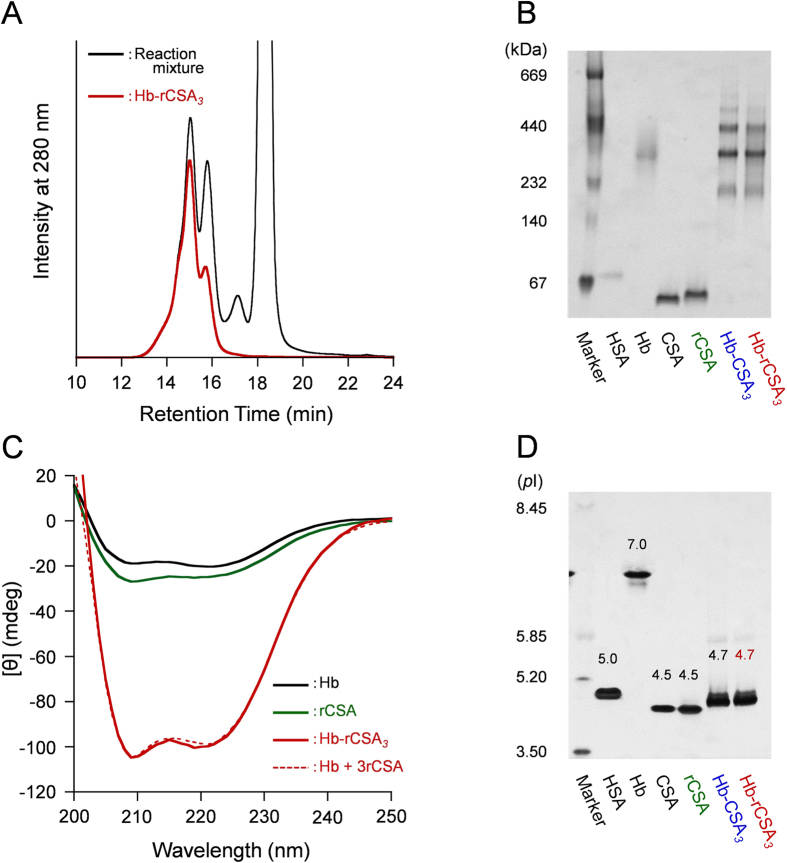
Physicochemical characterizations of Hb-rCSA_*3*_ cluster. (**A**) SEC profiles of reaction mixture of maleimide-activated Hb and rCSA (black line) and isolated Hb-rCSA_*3*_ cluster (red line). (**B**) Native-PAGE of Hb-rCSA_*3*_ cluster and related proteins. (**C**) CD spectra of Hb-rCSA_*3*_ cluster ([Hb] = 0.2 μM), Hb (0.2 μM), and HSA (0.2 μM) in PBS solution (pH 7.4) at 25 °C. The sum of Hb + 3rCSA spectrum coincided perfectly with that of Hb-rCSA_*3*_. (**D**) IEF patterns of Hb-rCSA_*3*_ cluster and related proteins. Full-length gel images of (**B,D**) are presented in Supplementary Fig. S5(A,B).

Succinimidyl-4-(*N*-maleimidomethyl)-cyclohexane-1-carboxylate (SMCC) was used as a cross-linker between the Cys-34 group of rCSA and the surface Lys groups of Hb (Fig. 3). First, SMCC was reacted with carbonyl Hb to produce maleimide-activated Hb. Site-specific binding to the free Cys-34 of rCSA and the maleimide-activated Hb yielded a core–shell cluster of Hb at the center and rCSA at the periphery. Later, SEC measurements of the reaction mixture depicted three peaks at the high molecular weight region (Fig. 4A, elution time 13–17 min). Native-PAGE also exhibited three new bands above rCSA (Fig. 4B and S5A). We collected all the large molecular products using gel filtration chromatography (GFC) and excluded the unreacted rCSA (Fig. 4A). Based on the total protein assay and Hb assay, the average rCSA/Hb ratio of the product was ascertained as 3.0 ± 0.2. This protein cluster comprises a mixture of Hb-rCSA_2_, Hb-rCSA_3_ (major), and Hb-rCSA_4_, which is indicated as Hb-rCSA_*3*_ with italicized subscript 3.

The CD spectrum of the Hb-rCSA_*3*_ cluster coincided accurately with the sum of the Hb spectrum and a three-fold amplified rCSA spectrum (Fig. 4C). This result proved that the secondary structure of individual protein unit was not perturbed by the coupling and that the rCSA/Hb composition ratio is 3/1 (mol/mol). The isoelectric point of Hb-rCSA_*3*_ cluster (*p*I: 4.7) was extremely close to the value of rCSA (*p*I: 4.5) (Fig. 4D and S5B), which supports enfolding of the Hb core by rCSAs. The surface net charges of this cluster are satisfactorily negative to circulate in the bloodstream for a long period. It is also noteworthy that the molecular features of Hb-rCSA_*3*_ cluster are exactly the same as those of the Hb-CSA_*3*_ cluster, which was prepared using native CSA.

### Immunogenicity

We analyzed immunochemical property of rCSA and Hb-rCSA_*3*_ cluster as an index of safety for administration into dogs. First, the immunological reactivity of Hb-rCSA_*3*_ cluster against anti-Hb antibody was examined. To perform simple and high sensitive detection of Hb, the immuno-chromatographic quick occult blood test kit was used. For this experiment, we synthesized a cluster bearing a human Hb (HbA) core, HbA-rCSA_*3*_. The HbA-antibody immune complexes migrated to the test area and were caught by immobilized rabbit anti-HbA antibody, generating sandwich complexes, which showed a red test-line signal (Fig. S6). In case of HbA-rCSA_*3*_ cluster, the test line was almost invisible. This means that the central HbA is covered by rCSAs and the anti-HbA antibody cannot recognize the epitope of the HbA molecule. Next, the immunological reactivity of Hb-rCSA_*3*_ cluster against anti-CSA antibody was evaluated using a latex agglutination immuno-assay. The absorption intensity of the reactant with rCSA or Hb-rCSA_*3*_ cluster demonstrated linear concentration dependence which coincided well with the calibration line of CSA (Fig. S7). All these results indicate that Hb-rCSA_*3*_ cluster does not bind anti-Hb antibody, but react with anti-rCSA antibody as same as CSA and rCSA.

### O_2_ Affinity and O_2_-Complex Stability

**Figure 5:**
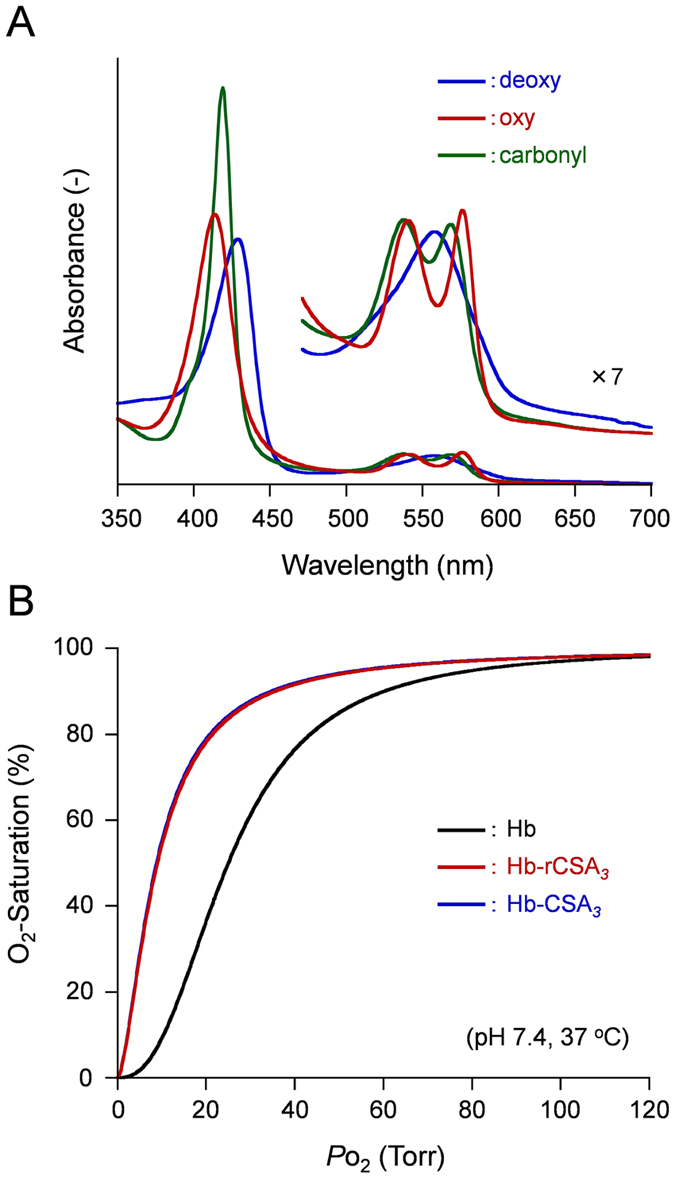
O_2_-binding to Hb-rCSA_*3*_ cluster. (**A**) UV-vis absorption spectra of Hb-rCSA_*3*_ cluster in deoxy, oxy, and carbonyl forms in PBS solution (pH 7.4) at 25 °C. (**B**) O_2_-equilibrium curves of Hb-rCSA_*3*_ and Hb-CSA_*3*_ clusters in PBS solution (pH 7.4) at 37 °C.

The visible absorption spectra of Hb-rCSA_*3*_ cluster in PBS solution (pH 7.4) equilibrated with N_2_, O_2_, and CO atmosphere (deoxy, oxy, and carbonyl forms) were fundamentally the same as those observed for Hb-HSA_*3*_, Hb-CSA_*3*_, and native Hb (Fig. 5A)^29^,^40^. The absorption maxima of these clusters showed good agreement, indicating that the wrapping of Hb with different albumins caused no alternation of electronic states of the prosthetic heme groups (Table S2).

The *P*_50_ of Hb-rCSA_*3*_ cluster was found to be 9 Torr (37 °C), whereas native Hb showed *P*_50_ of 23 Torr in the same experimental conditions (Fig. 5B, Table 1)^28^,^29^. The *n* value also decreased from 2.6 to 1.6. Covalent wrapping of Hb with rCSA caused *P*_50_ to decrease (O_2_ affinity to increase) 2.6-fold compared to naked Hb. Equivalent *P*_50_ and *n* reductions were also observed in Hb-CSA_*3*_ and Hb-HSA_*3*_ clusters (*P*_50_ = 9 Torr) (Table 1). The increase of O_2_ affinity and decrease in cooperativity of Hb-rCSA_*3*_ cluster was interpreted by the two structural alternations. First, the sulfhydryl group of Cys-93(*β*) in Hb was capped by the binding of free SMCC maleimide. Modification of Cys-93(*β*), which is a neighbor to the proximal histidine [His-92(*β*)] of the heme group^41^, is known to enhance O_2_ affinity^12^,^13^,^42^. Furthermore, it attenuates the possible motion of the *α*_1_*β*_1_/*α*_2_*β*_2_ interface of Hb^42^. Our detailed inspection revealed that the number of cysteinyl thiols per Hb decreased from 2.0 to 0.2 after the SMCC reaction, suggesting that two Cys-93(*β*) of Hb were reacted by SMCC maleimide. Fortunately, there is no Lys residue which can bind the opposite-terminal succinimide group of SMCC on Cys-93(*β*).

A second reason is the chemical modification of surface amino groups of Lys residues on Hb by SMCC. Such alternation is requisite for formation of the cluster, but modifications of Lys groups on Hb generally engender measureable changes of the O_2_ affinity and Hill coefficient^11^,^43^,^44^. Particularly, Lys-82(*β*) plays a crucially important role in controlling the quaternary structure deformation from the *Relaxed* state to a *Tense* state. We reported previously that Lys-82(*β*) is a binding partner of Cys-34 of HSA based on the 3D reconstruction of Hb-HSA_3_^28^. Therefore, it can be concluded that (i) the capping of Cys-93(*β*) elevates the O_2_ affinity and (ii) modification of surface Lys groups interferes with the Hb quaternary transition, resulting in the decline of O_2_-binding cooperativity.

Stability of the oxygenated Hb-rCSA_*3*_ cluster in PBS solution (pH 7.4) was examined using an autoxidation rate constant (*k*_ox_) of the Hb nuclei at 37 °C. The *k*_ox_ value of Hb-rCSA_*3*_ cluster was ascertained as 0.044 h^−1^, which was identical to the value of Hb-CSA_*3*_ cluster and similar to those of native Hb and Hb-HSA_*3*_^29^. The oxy Hb core in the cluster maintains good stability even after covalent binding with rCSA.

## Discussion

The rCSA and Hb-rCSA_*3*_ cluster shown in this work were designed for artificial blood products for exclusive use in the medical treatment of dogs. The rCSA has been prepared in a gram quantity using *Pichia* yeast expression. The protein showed identical features to those observed for native CSA. We first ascertained the crystal structure of rCSA at resolution of 3.2 Å, which revealed that the protein consists of three similar helical domains with 17 disulfide bonds. Although our purification protocol did not include a fatty acid removal process, which is typically a powder-charcoal treatment conducted under acidic conditions, the overall structure of rCSA resembled those of the defatted HSA and other mammalian albumin^36^,^39^. We used anion exchange chromatography at the last step of the purification. Thereby, anionic ligands in the protein might be eliminated, yielding a defatted rCSA.

Otagiri *et al*. examined the species differences of serum albumin with respect to drug binding properties, and demonstrated that warfarin (WF) and phenylbutazone (PBZ) cannot bind to CSA^45^. Commonly used drugs of many kinds with electronegative groups generally associate to the two primary binding sites of HSA, sites 1 and 2 located respectively in subdomains IIA and IIIA^3^. Both WF and PBZ are well-known site 1 binding drugs. However, ibuprofen, which is a typical site 2 binding drug, associates strongly to CSA with the same binding constant for HSA (*K* = 3.15 × 10^6^ M^−1^)^45^. Otagiri *et al*. reported that this difference might be attributable to a slight difference of the site 1 microenvironments of CSA and HSA. Curry *et al*. later reported comprehensive structural insight into HSA-drug complexes based on their numerous crystal structure data^38^. In the HSA-PBZ complex (defatted, PDB ID: 2BXC), the PBZ is accommodated in the center of the site 1 pocket with a single hydrogen bond interaction with the hydroxyl group of Tyr-150. Moreover, weak interaction through water molecules might exist with Lys-199 and Arg-222, which are located 4–6 Å from the edge of PBZ. The superposition image of rCSA and HSA-PBZ shows clearly that the architectures of their subdomains IIA (drug site 1) and IIIA (drug site 2) are identical (Fig. S3A). It is remarkable that the side chains of Tyr-150, Lys-199, and Arg-222 are located at the same positions (Fig. S3B). The reason why the PBZ does not bind to CSA could not be found from results obtained using the present resolution of our crystal structure analysis.

The only Cys with a free sulfhydryl group in rCSA was found to be Cys-34, which is responsible for the covalent binding to the surface Lys groups of Hb to create the core–shell structured Hb-rCSA_*3*_ cluster. The coupling of maleimide-activated Hb with rCSA advanced smoothly in the same way as the reaction with HSA or native CSA. Bovine Hb also possesses a free Cys at the 93 position of the *β* subunit^41^. Therefore, the maleimide-activated Hb might produce undesired Hb polymers. Nevertheless, such polymerization did not occur because the SMCC arm (10 Å spacer length) on the Hb is too short to make an intermolecular linkage with different Hb. The Cys-93(*β*) locates in hydrophobic cavities of Hb, at least 15 Å inside from the molecular surface^41^. Furthermore, the sulfhydryl groups of Cys-93(*β*) in Hb were mostly masked by the maleimide-terminal of excess SMCC. We inferred that the SMCC is an excellent cross-linker to synthesize a structurally defined core–shell Hb-rCSA_*3*_ cluster. This coupling procedure is likely to be applicable to preparations of different smart clusters composed of various combinations of an enzyme interior and serum albumin exterior.

The negative surface net charge of the Hb-rCSA_*3*_ cluster (*p*I: 4.7) proves the enfolding of Hb by rCSA. It can be favorable (i) to circulate in the bloodstream for a long time as an artificial O_2_-carrier and (ii) to avoid an undesirable vasopressor response by NO depletion. The immunochemical reactivity of Hb-rCSA_*3*_ cluster was markedly low against anti-Hb antibody, but was sufficiently high against anti-CSA antibody. We reasoned that the Hb center becomes a stealth protein by wrapping with HSAs, whereas the antigen epitopes of rCSA units are preserved after conjugation with Hb. These results suggest the immunological safety of Hb-rCSA_*3*_ cluster *in vivo*.

The O_2_ affinity of Hb-rCSA_*3*_ cluster became higher (*P*_50_ = 9 Torr) than the native Hb because the Cys-93(*β*) residues in Hb were blocked by the maleimide terminal of free SMCC. This high O_2_-affinity can be beneficial for an RBC alternate. Winslow *et al*. reported that the Hb-based O_2_-carrier with a low O_2_-affinity releases excessive O_2_ in the arterioles, causing autoregulatory vasoconstriction^18^,^46^. Intaglietta *et al*. demonstrated that RBCs having lower *P*_50_ (10 Torr) provide improvement of microvascular function in a hemorrhagic-shocked hamster model^47^. The high O_2_-affinity (lower *P*_50_) might be helpful to reduce arteriole O_2_-transport, and to eliminate cardiovascular side effects.

We reported previously the tissue distribution and metabolism of Hb-HSA_*3*_ cluster in rats^31^,^48^. The cluster and HSA were similarly distributed in the major organs at 1 h after injection. However, the accumulated quantity of the cluster was greater than that of HSA in the liver after 24 h. Rennen *et al*. demonstrated that the large-size proteins are cleared with predominant uptake by the liver^49^. Furthermore, we found that Hb-HSA_*3*_ cluster were metabolized in the body and excreted into urine, which is the same excretion pathway of HSA^48^. The Hb-rCSA_*3*_ cluster is likely to show high hepatic distribution after administration into dogs and to be excreted into urine.

We concluded that the Hb-rCSA_*3*_ cluster possesses three positive features for RBC substitute: (i) negative surface net charge, (ii) moderately high O_2_-affinity, and (iii) identical immunogenicity with CSA. Both rCSA and Hb-rCSA_*3*_ cluster can be of great medical importance as a potential plasma expander and O_2_-carrying fluid for veterinary blood transfusion in various clinical situations.

## Methods

### Materials and Apparatus

Bovine Hb was purified from fresh bovine blood purchased from Tokyo Shibaura Zouki Co. Ltd. Purification procedures of native CSA from canine plasma are presented in Supplementary Materials. All other reagents were purchased from commercial sources as special grades and were used without further purification. Deionized water (18.2 MΩ·cm) was prepared using water purification systems (Elix UV and Milli Q Reference; Millipore Corp.). SDS-PAGE and Native-PAGE were performed using an electrophoresis power supply (EPS 301; GE Healthcare UK Ltd.) and 5–20% polyacrylamide precast gradient gel (Hi-QRAS gel N; Kanto Chemical Co. Inc.). Isoelectric focusing (IEF) was conducted using an electrophoresis power supply (EPS 601; GE Healthcare UK Ltd.) and IEF protein gels (Novex pH 3–10; Thermo Fisher Scientific Inc.). The UV-visible absorption spectra were recorded using a UV-visible spectrophotometer (8543; Agilent Technologies Inc.) connected with a temperature control unit (89090A; Agilent Technologies Inc.). Circulation dichroism (CD) spectra were recorded using a spectrophotometer (J-820; Jasco Corp.). Mass spectra were obtained using a MALDI–TOF mass spectrometer (autoflex equipped with a pulsed N_2_ laser (337 nm); Bruker Daltonics K.K.). As the matrix, 0.1% sinapic acid, 0.05% trifluoroacetic acid, and 50% acetonitrile solution were used.

### Expression and Purification of rCSA

Complementary (c)DNA was synthesized via reverse transcription using canine liver RNA. The full-length cDNA of CSA was amplified by PCR using Prime STAR Max DNA polymerase (Takara Bio Inc.) and the following oligonucleotide primer set: forward [5′-TCGAAACGAGGAATTCATGAAGTGGGTAACTTTTATTT-3′] and reverse [5′-TGTCTAAGGCGAATTCTCAGACTAAGGCAGCTTGA-3′]. The amplified fragment was cloned into the *Eco*RI site of pHIL-D2 plasmid using an In Fusion reaction with Cloning Enhancer (Takara Bio Inc.), thereby generating pHIL-D2-CSA. Insertion of the entire CSA coding region was confirmed using DNA sequencing analysis. Then the obtained pHIL-D2-CSA plasmid vector was linearized by *Sal*I digestion and was used to transform the GS115 strain of *Pichia pastoris* (Thermo Fisher Scientific K.K.) by electroporation using an electroporator (MicroPulser; Bio-Rad Laboratories, Inc.).

Expression of rCSA was conducted by standard protocols provided by Thermo Fisher Scientific K.K. with some modifications. The transformed GS115 clone was first grown upon buffered mineral glycerol-complex (BMGY) medium (total 4 L) in a shaking incubator (Bio-Shaker G·BR-200; Taitec Corp.) with vigorous shaking (200 rpm) at 30 °C. When the culture medium reached an OD_600_ = 30–35, it was centrifuged (3000 × *g*, 10 min) to harvest the cells at 25 °C. To induce expression, the cell pellet was resuspended in a buffered mineral methanol complex (BMMY) medium (total 0.8 L). The expression was continued with vigorous shaking (200 rpm) at 30 °C for 10 days. During the cultivation, 100% methanol (1% volume of medium) was added every 24 h.

The secreted rCSA was isolated as follows. The growth medium was centrifuged (11,000 × *g*, 4 °C) to obtain the supernatant, which was brought to 50% saturation with ammonium sulfate at 25 °C. After leaving for 30 min at 4 °C, the slightly turbid solution was centrifuged (11,000 × *g*, 4 °C) to remove the dark brown precipitate. Subsequently, the supernatant fluid was brought to 95% saturation with ammonium sulfate and was stirred slowly for 30 min at 4 °C. The precipitated protein was collected by centrifugation (11,000 × *g*, 4 °C) and was dissolved in water. The brown solution was dialyzed against deionized water at 4 °C. Then 11% volume of 500 mM sodium phosphate (pH 5.8) was added. The resultant protein solution in 50 mM sodium phosphate (pH 6.0) was filtered using a membrane filter (C020A047A, 0.2 μm pore; Toyo Roshi Kaisha Ltd.).

Next, the obtained sample was loaded onto an affinity chromatography with a Toyopearl AF-Blue HC-650M (Tosoh Corp.) column. After washing with five-bed volumes of 50 mM sodium phosphate (pH 6.0), elution of rCSA was performed with 50 mM sodium phosphate (pH 7.4) containing 3 M NaCl. The eluent was dialyzed against deionized water at 4 °C, followed by addition of 25% volume of 100 mM Tris-HCl (pH 8.0). The resulting 20 mM Tris-HCl solution (pH 8.0) of the protein was filtered using a membrane filter (C020A047A, 0.2 μm pore). The sample was then applied to anion exchange chromatography with a Q Sepharose Fast Flow (GE Healthcare UK Ltd.) column using 20 mM Tris-HCl (pH 8.0) as the running buffer. After washing adequately with five-bed volumes of 20 mM Tris-HCl (pH 8.0) containing 100 mM NaCl, rCSA was eluted with 20 mM Tris-HCl (pH 8.0) containing 200 mM NaCl. The eluent was dialyzed against deionized water at 4 °C. Thereafter, 11% volume of 10× phosphate-buffered saline (PBS, pH 7.4) was added. Finally, the rCSA solution was concentrated to 30 mL using an ultraholder (Advantec UHP-76K; Toyo Roshi Kaisha Ltd.) with an ultrafilter (Advantec Q0100, 10 kDa MWCO; Toyo Roshi Kaisha Ltd.), and was sterilized with a sterile membrane filter (DISMIC-25CS, 0.2 μm pore; Toyo Roshi Kaisha Ltd.). All purification processes were followed by SDS-PAGE or Native-PAGE analysis. The concentration of rCSA was measured using a protein assay kit (Pierce 660 nm; Thermo Fisher Scientific K.K.). The cysteinyl thiol assay of rCSA was performed by reaction with 4,4′-dithiopyridine (4,4′-DTP), which binds the sulfhydryl group of the protein to generate 4-thiopyridinone (*λ*_max_: 324 nm)^50^.

### Crystallization, Data Collection, and Structure Determination of rCSA

The obtained rCSA was used directly for crystallization without further purification, such as powder-activated charcoal treatment. Colorless crystals (0.15 × 0.05 × 0.05 mm) grew spontaneously from a 30 mg/mL protein solution in the Morpheus screen condition H1 (Molecular Dimensions Ltd.) at 293 K within two weeks. The crystals were flash-frozen in liquid nitrogen. X-ray diffraction data were collected using an ADSC Quantum CCD detector with a Synchrotron radiation source at the Photon Factory AR-NW12A (Tsukuba, Japan). All data were collected at 100 K and were processed with HKL2000^51^. Data collection statistics are presented in Table S1. The rCSA structure was ascertained using molecular replacement with Auto-MR in PHENIX^52^. The structure of HSA with palmitic acid (PDB ID: 4BKE) was used as the search model^53^. In the initial electron density map, several helical features were clearly identified. Sequence assignment of the atomic model was conducted using the amino acid sequence of CSA^33^. Further iterative model building and refinement were performed using PHENIX and COOT^54^. For the poor electron density region (562–565), a poly-alanine model was built using COOT. Electron densities corresponding to the residues 1–2 (IA), 79–87 (IA-h4–h5), 114–120 (IA-h6–IB-h7), and 363–365 (IIB-h9–h10) were disordered in the model because of conformational flexibility at *N*-terminal, turns, and stretches of extended loops. Atomic coordinates of the rCSA structure reported in this paper have been deposited in the Protein Data Bank (PDB) under accession code 5GHK. All molecular images were produced using PyMOL (Schrödinger K.K.).

### Preparation of Hb-rCSA_*3*_

A DMSO solution of heterobifunctional cross-linker, succinimidyl-4-(*N*-maleimidomethyl)-cyclohexane-1-carboxylate (SMCC; Wako Pure Chemical Industries Ltd.) (20 mM, 0.2 mL), was added dropwise into PBS solution (pH 7.4) of carbonyl Hb (0.1 mM, 2 mL) in a round-bottom flask (20 mL volume). The mixture was stirred for 30 min in CO atmosphere at 4 °C. After removing unreacted excess cross-linker using gel filtration chromatography (GFC) with a Sephadex G25 (superfine) column (GE Healthcare UK Ltd.), the obtained SMCC-bound Hb (maleimide-activated Hb) was concentrated to 2 mL ([Hb] = 0.1 mM) using a centrifugal concentrator (Vivaspin 20 ultrafilter, 10 kDa MWCO; GE Healthcare UK Ltd.).

Before coupling of maleimide-activated Hb to rCSA, partially oxidized Cys-34 of rCSA was reduced using dithiothreithol (DTT). Aqueous DTT solution (10 mM, 0.1 mL) was added to the PBS solution (pH 7.4) of rCSA (1 mM, 2 mL) ([DTT]/[rCSA] = 0.5 mol/mol). Then the mixture was incubated for 1 h at 25 °C. The excess DTT was removed using GFC with a Sephadex G25 (superfine) column. The pooled eluent was condensed to 2 mL ([rCSA] = 1 mM) using a Vivaspin 20 ultrafilter (10 kDa MWCO). The increase of the mercapto-ratio of the Cys-34 was confirmed as ca. 80% using the 4,4′-DTP procedure^50^.

Then the maleimide-activated Hb solution (0.1 mM, 2 mL) was added slowly into rCSA solution (1 mM, 2 mL) in PBS, followed by stirring in CO atmosphere under dark conditions for 48 h at 4 °C. An aliquot of the reaction mixture was applied to size-exclusion chromatography (SEC) on an HPLC system (Prominence LC-20AD/CTO-20A/SPD-20A; Shimadzu Corp.) with an SEC column (YMC-Pack Diol-300 S-5; YMC Co. Ltd.) using 50 mM sodium phosphate (pH 7.4) as the mobile phase. The elution curve exhibited new triple peaks at the high molecular weight region. The reaction mixture was subjected to GFC with a Superdex 200 pg (GE Healthcare UK Ltd.) using PBS as the running buffer. All the fractions corresponding to the clusters were collected together. The entire protein concentration and Hb concentration were measured, respectively, using a protein assay kit (Pierce 660 nm) and Hb assay kit (Nescoat Hemokit-N; Alfresa Pharma Corp.). The average rCSA/Hb ratio of the product was estimated as 3.0 ± 0.2. We designated this cluster as Hb-rCSA_*3*_, with italicized subscript 3. The Hb-CSA_*3*_ cluster was prepared using the same procedure using native CSA obtained from canine plasma.

### Immunogenicity

Quick Chaser Occult Blood Test kit (Mizuho Medy Co., Ltd.), which detect human Hb (HbA) by immuno-chromatographic method, was used to evaluate the reaction between HbA-rCSA_*3*_ cluster and anti-HbA antibody. The HbA-rCSA_*3*_ cluster was synthesized by the same procedure as Hb-rCSA_*3*_ using HbA^30^. Two drops of the HbA-rCSA_*3*_ solution (0.1–1 μM) in PBS (pH 7.4) was applied to the assay plate and the appearance of red test-line signal was evaluated. Identical experiments with PBS, rCSA, and HbA were also conducted.

Immunological reactivity of HbA-rCSA_*3*_ cluster against anti-CSA antibody was measured using latex immuno-agglutination reagents (Canine albumin in urea; Shima Laboratories Co., Ltd.). The Hb-rCSA_*3*_ cluster solution ([rCSA unit] = 27–300 μg/mL, 30 μL) in PBS (pH 7.4) was first diluted with saline (120 μL), and aliquot (2.25 μL) of the solution was further diluted with special buffer (60 μL). Then the obtained sample solution was mixed with anti-CSA antibody sensitized latex (13 μL) at 37 °C, and the absorbance at 694 nm of the reactant was measured by a clinical chemistry analyzer (BioMajesty JCA-BM2250; Jeol Ltd.). Identical experiments with rCSA were also conducted.

### O_2_-Binding Property

To prepare the oxy Hb-rCSA_*3*_ cluster, the PBS solution (pH 7.4) of carbonyl Hb-rCSA_*3*_ ([Hb]: ca. 10 μM, 3 mL) in a round-bottom flask (20 mL volume) was rotated using a rotary evaporator and was exposed to 100% O_2_ gas under an ice-water bath. A liquid thin film formed on the inner wall of the flask was illuminated by a 500 W halogen lamp that was fixed above the rotating flask at a distance of 10 cm. After complete oxygenation, the oxy Hb-rCSA_*3*_ cluster solution was transferred to an optical quartz cuvette (10 mm path length) sealed with a rubber septum. To prepare deoxy Hb-rCSA_*3*_ cluster, N_2_ gas was blown to the O_2_-complex solution.

The O_2_ affinity (*P*_50_: O_2_-partial pressure where Hb is half-saturated with O_2_) and Hill coefficient (*n*) were obtained using an automatic recording system for O_2_-equilibrium curve (Hemox Analyzer; TCS Scientific Corp.) at 37 °C. The Hb-rCSA_*3*_ cluster in PBS solution (pH 7.4) was deoxygenated by flushing with N_2_ and was oxygenated by increasing the O_2_-partial pressure. The visible absorption spectra, *P*_50_, and *n* values of the Hb-CSA_*3*_ cluster were obtained using the same procedures.

### O_2_-Complex Stability

The O_2_-complex stability of the Hb-rHSA_*3*_ cluster was evaluated using the first-order autoxidation rate constant (*k*_ox_) of the core Hb. The oxy Hb-rCSA_*3*_ in PBS solution (pH 7.4, [Hb] = 10 μM, 2 mL) was put into an optical quartz cuvette (10 mm path length). The top of the cuvette was sealed with gas-permeable film (AeraSeal Film MAF710; Gel Co.), which facilitates air exchange but prevents water evaporation. The absorbance increase at 630 nm, which is based on metHb [Fe(III)-state] formation, was observed under aerobic conditions at 37 °C. After monitoring, the completely oxidized metHb-rCSA_*3*_ cluster was prepared by addition of small excess K_3_[Fe(CN)_3_]. From the absorption intensity change, the *k*_ox_ value was calculated using nonlinear least-squares curve fitting method. The O_2_-complex stability of Hb and Hb-CSA_*3*_ cluster was evaluated using the same procedure.

## Additional Information

**How to cite this article**: Yamada, K. *et al*. Artificial Blood for Dogs. *Sci. Rep.* **6**, 36782; doi: 10.1038/srep36782 (2016).

**Publisher’s note**: Springer Nature remains neutral with regard to jurisdictional claims in published maps and institutional affiliations.

## Supplementary Material

Supplementary Information

## Figures and Tables

**Table 1:** O_2_-binding parameters of Hb-rCSA_*3*_ and Hb-CSA_*3*_ clusters in PBS solution (pH 7.4) at 37 °C.

Hemoproteins	*P*_50_ (Torr)	*n* (−)	*k*_ox_ (h^−1^)
Hb†	23	2.6	0.037
Hb-rCSA_*3*_	9	1.6	0.044
Hb-CSA_*3*_	9	1.6	0.044
Hb-HSA_*3*_†	9	1.5	0.035

^†^Ref. 29.
