# Supplementary material for: Artificial Blood for Dogs
Source: Sci Rep. 2016 Nov 10;6:36782. doi: 10.1038/srep36782 (PMC5103191; doi:10.1038/srep36782)
Supplement: Supplementary Information [file srep36782-s1.pdf]

Supplementary Information

Artificial Blood for Dogs

Kana Yamada[1], Kyoko Yokomaku[1], Moeka Kureishi[1], Motofusa Akiyama[1], Kiyohito Kihira[2] & Teruyuki Komatsu[1]

[1] Department of Applied Chemistry, Faculty of Science and Engineering, Chuo University, 1-13-27 Kasuga, Bunkyo-ku, Tokyo 112-8551, Japan

[2] JEM Utilization Center, Human Spaceflight Technology Directorate, Japan Aerospace Exploration Agency (JAXA), 2-1-1 Sengen, Tsukuba-shi, Ibaraki 305-8505, Japan

Correspondence and requests for materials should be addressed to T.K. (e-mail: komatsu@kc.chuo-u.ac.jp)

Methods

Purification of Native CSA from Canine Plasma. Canine plasma was kindly provided from Kyoritsu Seiyaku Corp. To dissolve the frozen plasma, the sample was incubated in a refrigerator overnight at 4 °C. Then the plasma was centrifuged (10,000 × *g*, 4 °C) to remove the cryoprecipitate. The supernatant was brought to 50% saturation with ammonium sulfate at 25 °C. After leaving for 30 min at 4 °C, the solution was centrifuged (10,000 × *g*, 4 °C) and the supernatant was filtered using a membrane filter (DISMIC-25CS, 0.2 μm pore; Toyo Roshi Kaisha Ltd.). The obtained solution including CSA was dialyzed against deionized water at 4 °C. Then 11% volume of 500 mM sodium phosphate (pH 6.8) was added. The resultant solution in 50 mM sodium phosphate (pH 7.0) was filtered using a membrane filter (C020A047A, 0.2 μm pore; Toyo Roshi Kaisha Ltd.).

Next, the sample was loaded onto an affinity chromatography with a Toyopearl AF-Blue HC-650M (Tosoh Corp.) column. After washing with five-bed volumes of 50 mM sodium phosphate (pH 7.0), CSA was eluted with 50 mM sodium phosphate (pH 7.4) containing 3 M NaCl. The eluent was dialyzed against deionized water at 4 °C. Thereafter, 25% volume of 100 mM Tris-HCl (pH 8.0) was added. The resulting 20 mM Tris-HCl solution (pH 8.0) of CSA was filtered using a membrane filter (C020A047A, 0.2 μm pore). Then the sample was applied to anion exchange chromatography with a Q Sepharose Fast Flow (GE Healthcare UK Ltd.) column using 20 mM Tris-HCl (pH 8.0) as the running buffer. After washing with five-bed volumes of 20 mM Tris-HCl (pH 8.0) containing 100 mM NaCl, elution of CSA was performed with 20 mM Tris-HCl (pH 8.0) containing 300 mM NaCl. The eluent was dialyzed against deionized water at 4 °C, followed by addition of 11% volume of 10× phosphate-buffered saline (PBS, pH 7.4). Finally, the CSA solution was concentrated to 30 mL using an ultraholder (Advantec UHP-76K; Toyo Roshi Kaisha Ltd.) with an ultrafilter (Advantec Q0100, 10 kDa MWCO; Toyo Roshi Kaisha Ltd.), and was sterilized with a membrane filter (DISMIC-25CS, 0.2 μm pore). All the purification processes were followed by SDS-PAGE analysis. The concentration of CSA was measured using a protein assay kit (Pierce 660 nm; Thermo Fisher Scientific K.K.). The cysteinyl thiol assay of CSA was performed by reaction with 4,4'-dithiopyridine (4,4'-DTP), which binds sulfhydryl group of the protein to generate 4-thiopyridinone (λ_{max}: 324 nm)[50].

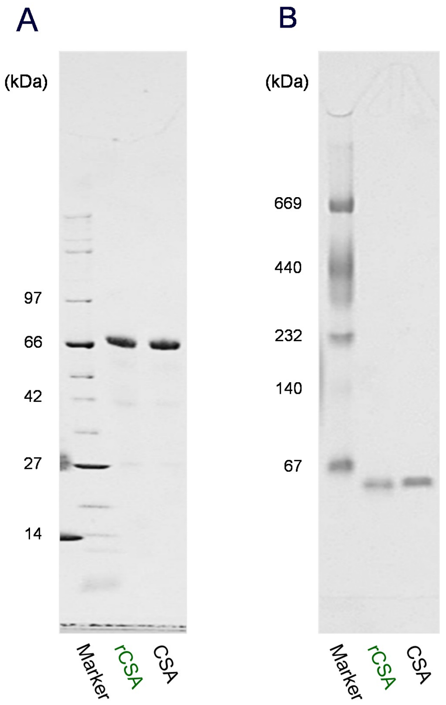

Figure S1. Full-length gel images corresponding to cropped images in Figure 1A (SDS-PAGE) and 1B (Native-Page).

	1	11	21	31	41
CSA	EAYKSEIAHR	YNDLGEEHFR	GLVLVAFSQY	LQQCPFEDHV	KLAKEVTEFA
HSA	DAHKSEVAHR	FKDLGEENFK	ALVLIAFAQY	LQQCPFEDHV	KLVNEVTEFA
	51	61	71	81	91
	KACAAEESGA	NCDKSLHTLF	GDKLCTVASL	RDKYGDMADC	CEKQEPDRNE
	KTCVADESAE	NCDKSLHTLF	GDKLCTVATL	RETYGEMADC	CAKQEPERNE
	101	111	121	131	141
	CFLAHKDDNP	GFPPLVAPEP	DALCAAFQDN	EQLFLGKYLY	EIARRHPYFY
	CFLQHKDDNP	NLPRLVRPEV	DVMCTAFHDN	EETFLKKYLY	EIARRHPYFY
	151	161	171	181	191
	APELLYYAQQ	YKGVFAECCQ	AADKAACLGP	KIEALREKVL	LSSAKERFKC
	APELLFFAKR	YKAAFTECCQ	AADKAACLLP	KLDELRDEGK	ASSAKQRLKC
	201	211	221	231	241
	ASLQKFGDRA	FKAWSVARLS	QRFPKADFAE	ISKVVTDLTK	VHKECCHGDL
	ASLQKFGERA	FKAWAVARLS	QRFPKAEFAE	VSKLVTDLTK	VHTECCHGDL
	251	261	271	281	291
	LECADDRADL	AKYMCENQDS	ISTKLKECCD	KPVLEKSQCL	AEVERDELPG
	LECADDRADL	AKYICENQDS	ISSKLKECCE	KPLLEKSHCI	AEVENDEMPA
	301	311	321	331	341
	DLPSLAADFV	EDKEVCKNYQ	EAKDVFLGTF	LYEYARRHPE	YSVSLLLRLA
	DLPSLAADFV	ESKDVCKNYA	EAKDVFLGMF	LYEYARRHPD	YSVVLLLRLA
	351	361	371	381	391
	KEYEATLEKC	CATDDPPTCY	AKVLDEFKPL	VDEPQNLVKT	NCELFEKLGE
	KTYETTLEKC	CAAADPHECY	AKVFDEFKPL	VEEPQNLIKQ	NCELFEQLGE
	401	411	421	431	441
	YGFQNALLVR	YTKKAPQVST	PTLVEVSRKL	GKVGTKCCKK	PESERMSCAE
	YKFQNALLVR	YTKKVPQVST	PTLVEVSRNL	GKVGSKCCKH	PEAKRMPCAE
	451	461	471	481	491
	DFLSVVLNRL	CVLHEKTPVS	ERVTKCCSES	LVNRRPCFSG	LEVDETYVPK
	DYLSVVLNQL	CVLHEKTPVS	DRVTKCCTES	LVNRRPCFSA	LEVDETYVPK
	501	511	521	531	541
	EFNAETFTFH	ADLCTLPEAE	KQVKKQTALV	ELLKHKPKAT	DEQLKTVMGD
	EFNAETFTFH	ADICTLSEKE	RQIKKQTALV	ELVKHKPKAT	KEQLKAVMDD
	551	561	571	581	
	FGAFVEKCCA	AENKEGCFSE	EGPKLVAAAQ	AALV	
	FAAFVEKCCK	ADDKETCFAE	EGKKLVAASQ	AALGL	

Figure S2. Comparison of amino acid sequences of CSA and HSA. The first row represents CSA sequence (colors correspond to the subdomain colors in Fig. 2A) and the second row represents HSA sequence (black)[33]. The yellow-marked amino acids are different kind pairs between CSA and HSA. The homology of these proteins is 79.8%.

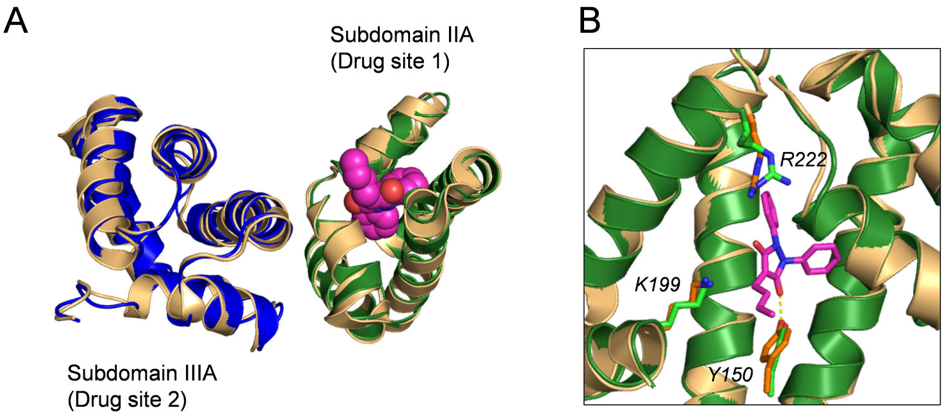

Figure S3. Superposition of crystal structures of rCSA and HSA-phenylbutazone (PBZ) complex. (A) Subdomain IIA (drug site 1: forest) and subdomain IIIA (drug site 2: blue) of rCSA are almost the same structures to the corresponding subdomains of HSA-PBZ complex (PDB ID: 2BXC, light orange)[38]. PBZ is depicted in space-filling representation and colored in light magenta. (B) PBZ (light magenta) bound in the hydrophobic pocket of drug site 1 of HSA (light orange) (PDB ID: 2BXC). The PBZ forms a hydrogen bond with Tyr-150 of subdomain IB. Lys-199 and Arg-222 may form weak interactions with PBZ through water molecule. The α-helices of rCSA is colored in forest. The positions and orientations of the amino acid residues (Y150, K199, R222) of HSA (orange) and rCSA (green) are almost identical.

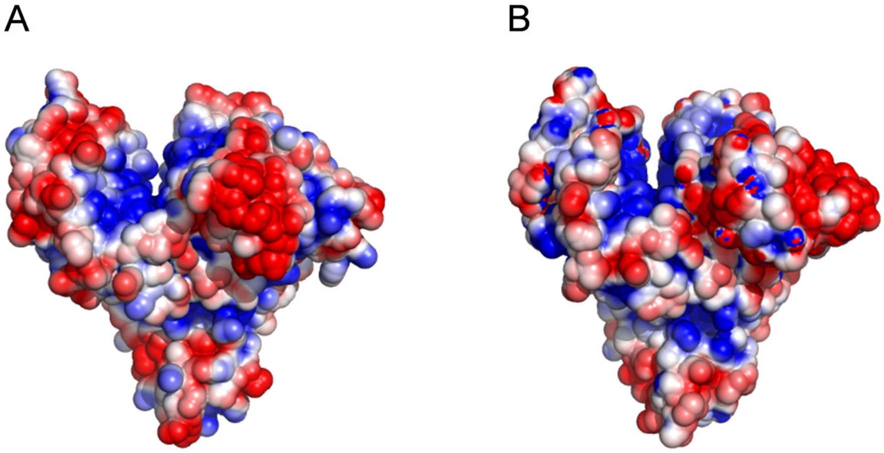

Figure S4. Surface electrostatic potential representations of (A) rCSA and (B) HSA. Blue and red respectively represent positive charge and negative charge density. Calculations were carried out using Adaptive Poisson-Boltzmann Solver (APBS) and PyMOL. The pdb files were converted to pqr files for APBS electrostatics calculations by PDB2PQR service[S1]. PDB ID of HSA: 1E78[36].

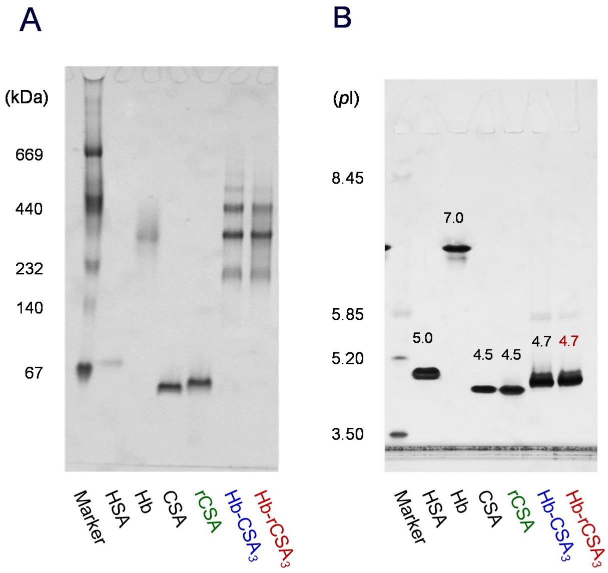

Figure S5. Full-length gel images corresponding to cropped images in Figure 4B (Native-PAGE) and 4D (IEF).

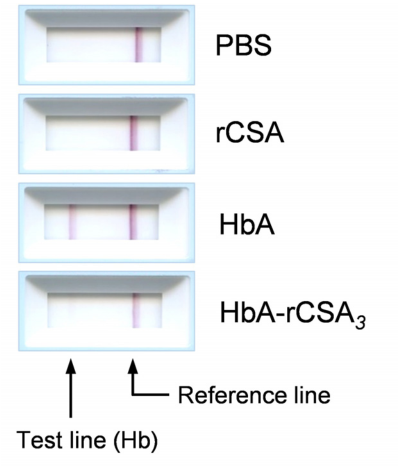

Figure S6. Immunological reactivity against anti-HbA antibody. The result of quick chaser occult blood test kit. Appearance of a red test-line indicates the immunological activity to anti-HbA antibody.

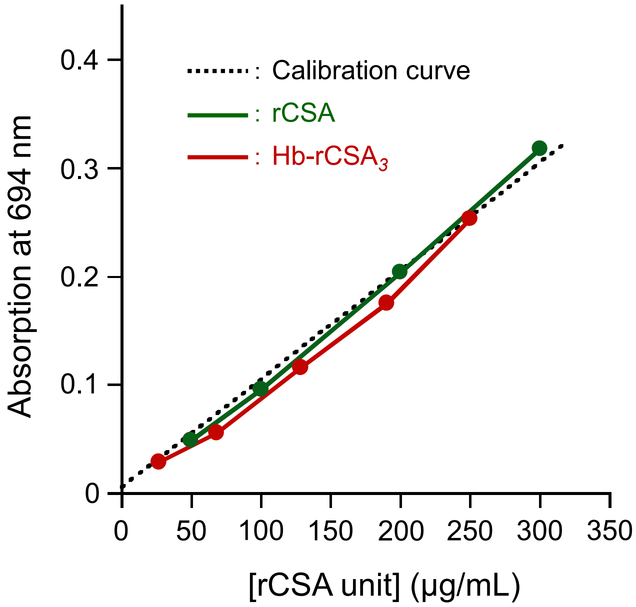

Figure S7. Immunological reactivity against anti-CSA antibody. The relation between CSA unit concentration of the sample and absorption intensity of the reactant solution at 694 nm. The dotted line is calibration curve prepared using CSA standard.

Table S1. X-ray crystallography data collection and refinement statistics of rCSA

Wavelength (Å)	1.0000
Resolution range (Å)	34.84–3.0 (3.05–3.0)
Space group	P 1 21 1
Cell dimensions	a = 46.72 Å, b = 118.4 Å, c = 58.33 Å
	α = 90°, β = 114.82°, γ = 90°
Total reflections	77559
Unique reflections	11596 (577)
Multiplicity	6.7 (6.0)
Completeness (%)	99.9 (99.8)
Mean I/sigma (I)	30.3 (6.6)
B-factor ($Å^2$)	48.12
R-merge	0.110 (0.453)
Mosaicity Range (º)	1.25–1.98
**Refinement**	
Resolution range (Å)	34.84–3.2 (3.315–3.2)
No. reflections	9425 (948)
R-work	0.2530 (0.3006)
R-free	0.2916 (0.3322)
No. non-hydrogen atoms	4424
Macromolecules	4424
RMS (bonds)	0.008
RMS (angles)	0.87
Ramachandran favored (%)	90
Ramachandran outliers (%)	1.6
Clashscore	9.47
Average B-factor	47.80

Statistics for the highest-resolution shell are shown in parentheses.

Table S2. UV-vis absorption spectral data of Hb-rCSA*3* and Hb-CSA*3* clusters in PBS solution (pH 7.4) at 25 °C

	λ_{max} (nm)		
Hemoproteins	deoxy	oxy	carbonyl
Hb-rCSA*3*	429, 556	414, 541, 577	419, 538, 569
Hb-CSA*3*	429, 556	414, 541, 577	420, 538, 569
Hb-HSA*3*	430, 556	413, 541, 577	420, 538, 569
Hb [†]	430, 555	414, 541, 577	420, 538, 569
HbA [‡]	430, 555	415, 541, 577	419, 540, 569

[†] From ref. 29. [‡] Ref. 40.

Reference

S1. Dolinsky, T. J., Nielsen, J. E, McCammon, J. A. & Baker, N. A. PDB2PQR: an automated pipeline for the setup, execution, and analysis of Poisson-Boltzmann electrostatics calculations. *Nucleic Acids Res.* **32**, W665–W667 (2004).
